# Origins of violence: evolutionary decoupling between mild and lethal conspecific aggression in primates

**DOI:** 10.1093/evlett/qrag002

**Published:** 2026-03-03

**Authors:** Bonaventura Majolo, Samantha J Wakes, Marcello Ruta

**Affiliations:** School of Psychology, Sport Science & Wellbeing, University of Lincoln, Lincoln, United Kingdom; School of Psychology, Sport Science & Wellbeing, University of Lincoln, Lincoln, United Kingdom; School of Natural Sciences, University of Lincoln, Lincoln, United Kingdom

**Keywords:** conflict, conspecific aggression, infanticide, killing, violence

## Abstract

Theories on the evolutionary origins of human aggression have often implicitly assumed that conspecific aggression is a single behavioral trait. However, different types of aggression can be described, based upon their intensity, frequency, as well as the age and sex of the opponents. The phylogenetic relationships between different types of aggression remain poorly understood. We tested the strength of correlated evolution between five distinct types of aggression in primates, namely, between- and within-group mild (i.e., not life-threatening) aggression, between- and within-group adulticide, and infanticide. We collected data on 100 free-ranging, non-provisioned and group-living species, including humans. Phylogeny had a weaker effect on mild than on lethal aggression; the effect of phylogeny was greater for adulticide, especially when we partitioned our analyses by the sex of the attacker. Furthermore, we found a positive correlation between within- and between-group adulticide, and with infanticide; these results were mostly confirmed when we considered the sex of the attacker. Conversely, the two types of mild aggression were weakly related with lethal aggression. Our study highlights the importance of treating aggression as a complex set of interrelated traits in comparative analyses. Our findings indicate that mild aggression is not closely linked to killing; thus, the escalation of aggression may follow more complex patterns that what predicted by current socio-ecological models.

## Introduction

One of the most contentious and enduring debates, at the intersection of many disciplines, such as evolutionary biology and anthropology, concerns the origins of human aggression. Specifically, the debate focuses on whether aggression is deeply rooted in our evolutionary past or whether it results from cultural and/or socio-ecological changes that have occurred in human prehistory, including the emergence of agriculture and state-level societies (Fry, 2006; Kelly, 2013; Majolo, 2019; Zefferman & Mathew, 2015). This debate has long revolved around the implicit and as yet untested assumption that conspecific aggression is a single trait, one that humans either possess or lack as an inherited trait, irrespective of the number, identity, and characteristics of the opponents, as well as the intensity and contextual factors of aggressive encounters. One reason this assumption has remained untested is the inherent difficulty of reliably inferring rates and forms of aggression from archaeological records (Ferguson, 2013; Kim & Kissel, 2018). Comparative analyses of aggression across primates can yield valuable insights into the evolutionary origins and diversification of aggression (Gómez et al., 2021; Manson et al., 1991; Willems et al., 2013).

Conspecific aggression is a key component of social interactions in virtually all animal species, as it brings fitness costs and benefits and affects social evolution (Bowles, 2012; Silk, 2014; Wilson et al., 2014). The repertoire of behaviors expressed during aggressive interactions (e.g., facial or vocal displays), the sex exhibiting a greater proclivity for aggression, and the number of individuals involved, all vary markedly across taxa, and ecological and social contexts (Newton-Fisher & Thompson, 2012). Moreover, aggression can be described according to the age of the opponents and, among group-living species, to whether opponents belong to the same social group or to distinct groups (Majolo et al., 2005; Manson et al., 1991; Willems et al., 2013).

A key factor of conspecific aggression is its intensity, which ranges from mild to lethal (Newton-Fisher & Thompson, 2012; Wilson et al., 2014). The majority of aggressive interactions are mild (Isbell, 1991; Wheeler et al., 2013; Willems & van Schaik, 2015) and rarely result in life-threatening injuries (Majolo et al., 2020; Wheeler et al., 2013). Lethal aggression is less frequently observed (Manson et al., 1991). Conspecific killing occurs either through accident or as a deliberate act, and its likelihood is affected by differences in fighting capacity between opponents, e.g., disparity in physical strength or in the number of individuals comprising antagonist parties (Wrangham, 1999). For the attacker, the potential costs of killing a conspecific adult (Lane & Briffa, 2017) may explain why infanticide appears to be more common than adulticide. Male infanticide is often observed in species where males are considerably larger than females and/or that form bachelor male groups that can overpower both parents (Lukas & Huchard, 2014; Opie et al., 2013). However, while adulticide can permanently eliminate competitors, it typically involves direct physical confrontation which carries a significant risk of serious injury or death for both the aggressor and the target individual (Silk, 2014; Wrangham, 1999). For these reasons, adulticide is more likely to occur when the attackers significantly outnumber the victim (Wilson et al., 2014; Wrangham, 1999).

Despite the voluminous literature on the evolution and socio-ecology of conspecific aggression (Manson et al., 1991; Silk, 2014; Sterck et al., 1997; Wrangham, 1999), our understanding of whether, and to what extent, different types of aggression are phylogenetically correlated to one another is still limited. For example, an evolutionarily informed analysis of conspecific killing across vertebrates found that primates are more violent than other taxa (Gómez et al., 2016). However, the study in question combined data on infanticide, cannibalism, and adulticide, even though these three types of lethal aggression have distinct functions and involve individuals of different age and group membership. In contrast, a comparative phylogenetic study of aggression in mammals, that distinguished among different categories of lethal aggression (Gómez et al., 2021), found a correlation between female adulticide and infanticide, but not between male adulticide and infanticide.

Phylogenetic comparative data hold considerable promise for our understanding of social evolution and the origins of human aggression (Fry, 2006; Majolo, 2019; Wrangham, 2018). Quantifying the strength of correlations among distinct types of aggression would enhance the accuracy of socio-ecological and animal contest models. For example, classical socio-ecological models (Isbell, 1991; Sterck et al., 1997) incorporate predictions on the intensity of competition, but do not explicitly address whether, and under which species-specific, ecological, and individual-level conditions, mild aggression—whether between- or within-groups—is likely to escalate to killing. Due to their distinct functions, payoffs and occurrence rates (Isbell, 1991; Lukas & Huchard, 2019; Majolo et al., 2020; Manson et al., 1991; Wheeler et al., 2013), different types of aggression may be hypothesized to exhibit a weak correlation. At the same time, some types of aggression may be triggered by identical socio-ecological conditions (Isbell, 1991) and/or share the same physiological or cognitive processes (Dixson, 1980; Wrangham, 1999). For example, mild aggression might be evolutionarily related to killing, if species that frequently engage in mild aggression are also more likely to participate in numerically asymmetric competitive interactions (Aureli et al., 2006; Wrangham, 1999). Alternatively, in those species that face intense between- and within-group competition, killing competitors from other groups may yield fitness benefits, such as territorial gains (Lemoine et al., 2020). In those same species, adulticide within the group may be too costly, because reducing group size negatively affects the chances of outcompeting neighbor groups.

Acknowledging the complex and multifaceted nature of aggression, we set out to investigate the relationships between five types of aggression across group-living primates (including humans), namely: between- and within-group mild aggression, between- and within-group adulticide, and infanticide. In those cases where correlations among different types of aggression were identified, we also assessed whether such correlations changed when we split aggressive behaviors by sex of the attacker. We focus on primates to exploit the extensive body of literature on aggression in this taxon and because of its relevance to our knowledge of the evolutionary factors underpinning human social evolution (Manson et al., 1991; Opie et al., 2013).

## Material and methods

### Data collection

We extracted data from published and unpublished studies to assemble our dataset on aggression in 100 primate taxa (98 species and two pairs of subspecies; see below), inclusive of 43 genera and 11 primate families (the dataset is available here: https://hdl.handle.net/10779/lincoln.31026160). To enable comparison between studies and across species, we adopted definitions of the five types of aggression (Table 1) that were not unique to the behavioral repertoires of individual species. Our initial plan was to obtain data on the rate of occurrence for all five aggression types. However, we had to rely on binary categories (presence/absence) for the three types of lethal aggression (Table 1) due to a lack of more detailed data. We excluded studies conducted on captive, semi-free ranging, or provisioned groups, as their behavior patterns can differ to those of their wild counterparts (Hosey, 2005). Furthermore, we only considered group-living species, in order to discern patterns of between- and within-group aggression. To this end, we included species living in groups with a stable composition through time and sharing a common ranging area, rather than a loose association around resources (Dunbar et al., 2018).

**Table 1:** Definitions and unit of measurements of the five types of aggression.

Aggression Type	Definition
Between-group mild aggression	We used the definition of between-group encounter used by each study included in the dataset, and we excluded long-distance between-group interactions through territorial advertisement and loud calls. A between-group encounter was defined to involve mild aggression when a minimum of one adult individual displayed one or more of the following behaviors toward members of the opposing group: aggressive calls, bodily and/or facial expressions given/displayed by an individual toward one or more members of the opposing group, chases, pushes, hits, or bites not resulting in life-threatening injuries (27). Unit of measurement: proportion of mildly aggressive between-group encounters (N of encounters involving mild aggression/total N of encounters recorded during the course of each study).
Within-group mild aggression	Number of mild aggressions (see definition above) given by one or more adult individual toward another member of the group. Unit of measurement: Frequency of within-group mild aggression (average N of mild aggression given per individual/hour).
Between-group and within–group adulticide	Killing of one or more adults by one or more adults from within the same or from a different social group than the victim(s). We considered adulticide to have occurred, at least once, in a species/sex, when lethal aggression was observed (i.e., directly witnessed by researchers) or there was strong evidence to infer that lethal aggression had occurred (i.e., the attack was observed and the victim disappeared after the attack, but the victim’s body was never found). In four datasets, we excluded cases in which lethal aggression was suspected but not observed, but we included suspected cases of adulticide in the “Binary1” dataset (see Methods and Supplementary Material Table SM1). Unit of measurement: binary—adulticide occurs in the species/sex (at least once) or not.
Infanticide	Killing of an infant by a conspecific adult. Similarly to adulticide (above), we included cases where infanticide was observed or there was strong evidence to infer that it had occurred. Unit of measurement: binary—infanticide occurs in the species (at least once) or not.

We reviewed the primatological literature, published between 1950 and June 2022, using Google Scholar (http://scholar.google.com) for scientific papers and academic books, and ProQuest (https://about.proquest.com/en) for dissertations and theses. We used the following keywords for the literature review: “kill,” “violence,” “death,” “died,” “fatal,” “attack,” “lethal,” “infanticide,” “aggression,” “intra-group,” “inter-group,” “between-group,” “within-group,” and “competition.” We entered these keywords in various combinations to fully review the primate literature. Moreover, we designed a short survey (Supplementary Material SM1.2) utilizing Qualtrics software (https://www.qualtrics.com) to include unpublished data in our study. We advertised this survey on social media, we shared it with members of primatological societies, and we contacted individual researchers who worked on species for which we had data on at least one, but not on all the five aggression types. Contacting individual researchers somehow biased our search for unpublished data, but substantially increased response rate and reduced the number of taxa that had to be excluded due to missing data. Thirty-nine researchers completed the survey, providing data on 28 species/sub-species.

For female and male adulticide, we used the data from Gomez et al. (2021), which we divided into between- and within-group (female/male) adulticide, using the original studies cited in the paper (since Gomez and colleagues did not differentiate adulticide in relation to the group membership of the opponents). We critically evaluated these data in light of more recent publications and of the unpublished data from the survey. Through these data, we identified seven species that have been observed to display adulticide but that were recorded as “adulticide absent” in Gomez et al. (2021).

For female and male infanticide, we extracted the majority of data from Lukas & Huchard (2014, 2019; 66 taxa = 66% of data) and from Opie et al. (2013; 13 taxa). For the remaining 21 taxa, we entered new data from published or unpublished studies; these data included 9 taxa that were present in either Lukas & Huchard (2014, 2019) or Opie et al.’s (2013) datasets, but for which new data on infanticide were published afterward.

The two types of mild aggression were often reported using different units of measures across studies, and data on more than one population/group were available for some of the species in our dataset. For these two aggression types, we used the units of measurement (Table 1) that were most frequently reported in the literature and to which other units of measures could be converted to (see Supplementary Material for details on the conversions used). When we had more than one datum, on a species in our dataset, that met our inclusion criteria, we calculated the average value for that species and type of mild aggression. In order to maximize the number of taxa represented in our dataset, we included qualitative data on mild aggression, as done in previous comparative analyses (Willems et al., 2013). Since this approach potentially introduces noise to the data, we built five different datasets on the five aggression types, each using a different method to calculate species-specific data on aggression (see further details on this procedure in Supplementary Material Table SM1). In the “Baseline” dataset we used averages for mild aggression. In two other datasets (the “High” and “Low” datasets, respectively), we used the highest and lowest values for mild aggression, within the range identified from the qualitative and quantitative data. Finally, in the last two datasets (“Binary1” and “Binary2”), we converted the data for mild aggression into binary variables (absence = 0, for species with a proportion of mildly aggressive encounters or with a frequency of within-group mild aggression <0.50; presence = 1 for species with a value of mild aggression ≥0.50). The data for the three types of lethal aggression remained the same in four datasets. However, in the “Binary1” dataset, we included suspected cases of lethal aggression (as done by Gomez et al., 2021) to analyze whether our conservative approach (that did not include suspected cases; Table 1) had an effect on our results. Therefore, in the “Binary1” dataset, two additional species displayed between-group adulticide and one species within-group adulticide, whereas these species were coded as “adulticide absent” in the other four datasets.

We added to our datasets species-specific information on five socio-ecological variables that are known to influence one or more forms of aggression, namely: sexual dimorphism (Gómez et al., 2021; Heldstab et al., 2021; Plavcan & Van Schaik, 1997), within-group sex ratio (Surbeck et al., 2017; Willems et al., 2013), group size (Cowl & Shultz, 2017; Willems & van Schaik, 2015), proportion of leaves in the diet (Isbell, 1991; Powell et al., 2017; Sterck et al., 1997; Wheeler et al., 2013), and (only for between-group mild aggression and adulticide) degree of territoriality (Kappeler & van Schaik, 2002; Mitani & Rodman, 1979; Willems et al., 2015). We quantified sexual dimorphism as the natural logarithm of the ratio between the body weight of adult males and females (Smith, 1999; Willems et al., 2013), and intragroup sex ratio as the number of adult males in the group divided by the number of adult females. For group size, we considered the total number of adults, sub-adults, and juveniles comprising the group. Following (Grabowski et al., 2023), we treated the proportion of leaves in the diet as a binary variable (0 = species with <0.5 of leaves in their diet; 1 = species with ≥0.5). Finally, we used the D-index, namely the ratio between average daily traveled distance and home range size (Mitani & Rodman, 1979), to estimate the degree of territoriality of a species. We extracted data on sexual dimorphism in 88 taxa from Heldstab et al. (2021) and Gomez et al. (2021), and data on within-group sex ratio in 80 taxa from Willems et al. (2013). We extracted data on group size in 96 taxa from Powell et al. (2017), DeCasien et al. 2017, and Willems et al. (2013, 2015), data on diet (proportion of leaves) in 90 taxa from Powell et al. (2017) and Grabowski et al. (2023), and data on the D-Index (degree of territoriality) in 79 taxa from Willems et al. (2013, 2015). The data on these five variables for the remaining taxa were mostly extracted from (Rowe & Myers, 2016).

### Phylogenetic tree construction

A set of 1,000 time-scaled, Bayesian phylogenies of primates were sampled at random from the VertLife website (Upham et al., 2019). These phylogenies were subsequently loaded into R version 4.0.2 (R Foundation for Statistical Computing, Vienna, Austria., 2020), using the package *ape* (version 5.4.1; Paradis & Schliep, 2019). We extracted pair-wise distances between trees in the R package *phangorn* (Schliep, 2011), using the branch score distance as the preferred distance metric, calculated as the square-root of the sum of squared differences between the branch lengths of a pair of trees (Kuhner & Felsenstein^1994^. For all subsequent analyses, we chose a tree from the random sample as follows: we computed a matrix of pairwise tree-to-tree distances and summed the distances between each tree and all others (using the colSums function in base R). The tree with the lowest total distance—i.e., the minimal cumulative divergence from all other trees—was selected for subsequent analyses and pruned to retain only the species included in our dataset (drop.tip function in *ape*). We manually added subspecies to the topology thus obtained (*Pan troglodytes schweinfurthii* and *P.t. verus*, and *Piliocolobus badius temminckii* and *P.b. badius*), using the bind.tip function in *phytools* (Revell, 2012). We estimated the position of these subspecies, relative to their immediate sister taxon, at 0.93 million years divergence from the present for *P.t. schweinfurthii* and *P.t. verus* (Stone et al., 2010), and at 0.25 million years for *P.b. temminckii* and *P.b. badius* (Ting, 2008).

### Statistical analyses

We ran a series of Bayesian generalized linear mixed models (BGLMMs) with the “brms” (Bürkner, 2017a) and “rstan” (version 2.32.6; (Stan Development, 2024) packages in R version 4.0.2. Below, we present the results of the BGLMMs run with the “Baseline” dataset, since it contains average values for mild aggression for each species, which is the most common approach for comparative analyses (e.g., Wheeler et al., 2013; Willems et al., 2013); we present the results on the other four datasets in the Supplementary Material (Table SM2)

We used two approaches to analyze the relationship between the five aggression types at the species level (i.e., species-BGLMM) and divided by the sex of the attacker (i.e., sex-BGLMM). First, we ran a null BGLMM which included the five aggression types and phylogenetic relatedness, through the inclusion of species ID as a random effect, weighted by the variance–covariance matrix calculated from the Bayesian consensus tree. For the null BGLMM, we report, for each type of aggression, the Bayesian equivalent of the conditional *R*^2^ statistic (plus its error, and the lower and upper 95% credible intervals: 95% CIs), which is a measure of the proportion of the total variability accounted for by the random effect, i.e., phylogeny. Second, we ran a full BGLMM which included the five aggression types, phylogenetic relatedness (i.e., species ID as a random factor), and the five socio-ecological variables as controls. We used the full BGLMM to control for the possibility that the relationship between the five aggression types was modulated by socio-ecological factors that are known to affect one or more types of aggression (see above). For example, adult males face a lower risk, when attacking infants and adult females, in species with a male-biased than in species with female-biased dimorphism; thus, a positive relationship between male adulticide and infanticide might only emerge in species with a male-biased sexual dimorphism. In the full BGLMM, we z-transformed and centered the four continuous socio-ecological variables (excluding proportion of leaves), using the “scale” function in R (Bürkner, 2017a; Schielzeth, 2010).

For each model, we estimated the model parameters by running four Markov Chain Monte Carlo (MCMC) chains in parallel, each for 6,000 iterations (with 3,000 post-warm-up samples retained), resulting in a total of 12,000 posterior draws. We used various methods to assess whether our models effectively explained the relationship between the five types of aggression. We visually inspected the posterior predictive plots to assess if the observed data were distributed within the fitted models. We used the *R*-hat values (i.e., the potential scale reduction factors, which measure between-chain variability) to analyze the convergence of the MCMC-chains, with the assumption that *R*-hat values close to 1, but ≤1.01 indicate convergence of the chains (Bürkner, 2017b). Finally, we used trace and density plots for the parameters in each model to ensure that the chains were well-mixed and stationary (Bürkner, 2017b), and that the models achieved convergence. All the models presented in our manuscript passed these checks.

For each pairwise relationship between two types of aggression, we present the estimate and its error, the 95% CIs, and the Tail Effective Sample Size, which measures the sampling efficiency of the posterior distribution (Vehtari et al., 2021). We used the magnitude of the estimate, its 95% CIs, and the percentage of the posterior distribution that is in the same direction of the mean to interpret the strength of the phylogenetic relationship between two types of aggression; we considered, as biologically relevant, correlations where ≥90% of the posterior distribution had the same sign of the mean (McElreath, 2020). For brevity, we present the statistics for the socio-ecological controls in the supplementary material, and we do not discuss their effect in the main text.

In the two species-BGLMMs (null and full models), we analyzed the evolutionary relationship between the five types of aggression. We specified a Bernoulli distribution for the three types of lethal aggression (between- and within-group adulticide, and infanticide) and a zero-one inflated beta and a hurdle gamma distribution for, respectively, between- and within-group mild aggression.

Since the two species-BGLMMs showed a moderate/strong evolutionary relationship between within- and between-group adulticide and infanticide (see results), we ran two sex-BGLMMs to analyze whether the strength of this relationship was confirmed when considering the sex of the attacker. The two sex-BGLMMs contained six types of lethal aggression (i.e., between- and within-group adulticide, and infanticide, each split by the sex of the attacker—male or female), as response variables. We did not analyze sex of the attacker for between- and within-group mild aggression, because in the species-BGLMMs, we found weak evolutionary relationship involving these two types of aggression (see results). Moreover, dividing data for these two types of aggression by sex would result in a substantially smaller number of species, due to gaps in the literature on sex-specific mild aggression. Thus, we prioritized testing the same set of species to obtain comparable results across models, instead of having a different number and representation of taxa in the models.

## Results

In our dataset, 22% of the species displayed between-group adulticide (by females: 9% of species; by males: 22% of species), 19% within-group adulticide (by females: 5% of species; by males: 17% of species), and 65% species displayed infanticide (by females: 16% of species; by males: 61% of species). The mean proportion of mildly aggressive between-group encounters (± SD) was 0.52 ± 0.36, and mean frequency of within-group mild aggression per hour was 0.17 ± 0.27.

**Figure 1:**
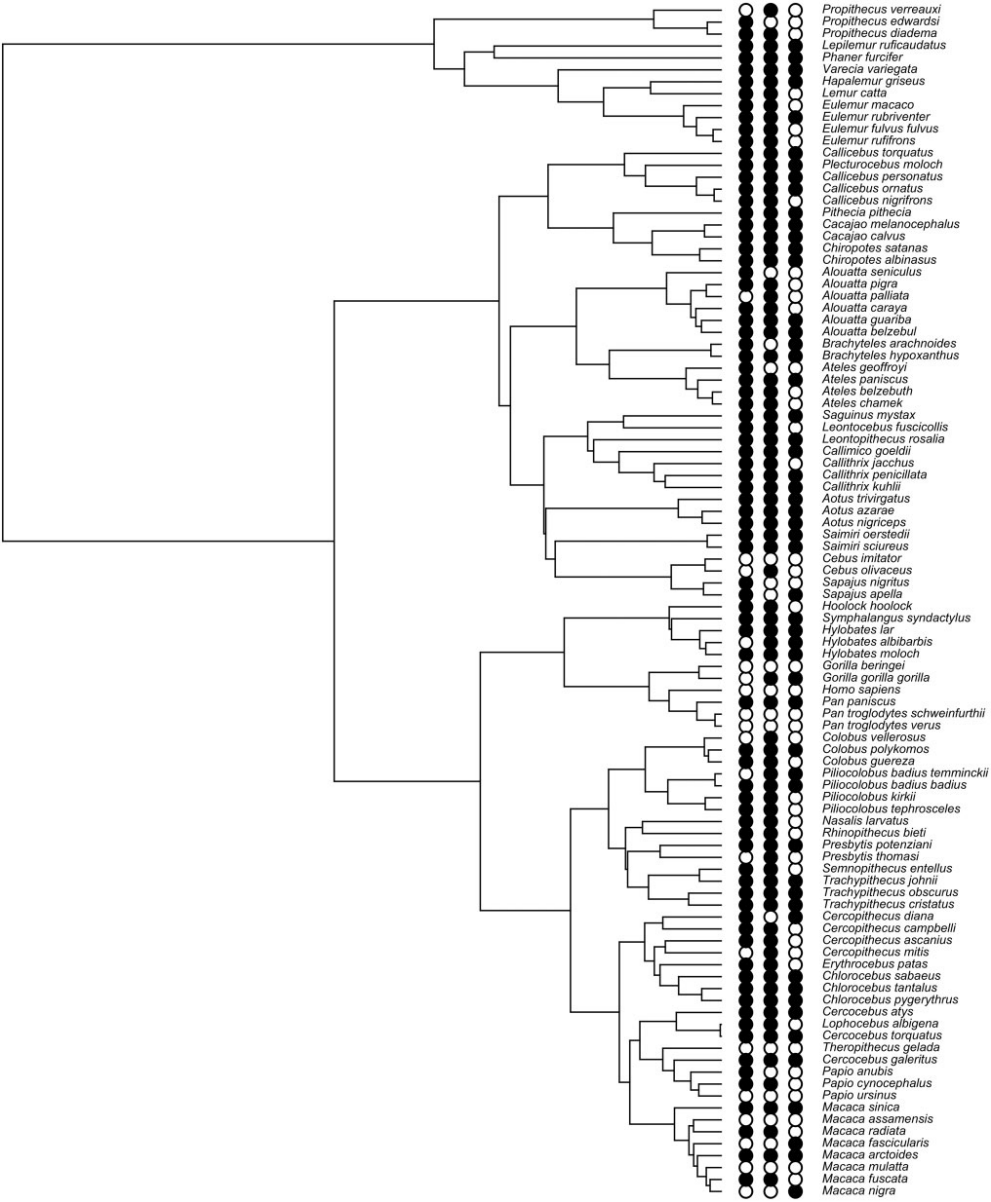
Phylogenetic tree of the taxa included in our dataset, showing the presence/absence (empty/full circles, respectively) for the three types of lethal aggression, namely between-group adulticide (left column), within-group adulticide (middle column) and infanticide (right column).

In the null species-BGLMM, phylogeny explained a small amount of variance for the five types of aggression; infanticide had the largest effect of phylogeny (Table 2). We found moderate and positive evolutionary correlations between the three types of lethal aggression, particularly between within- and between-group adulticide (Table 3 and Figure 1). Conversely, we found weak correlations involving the two types of mild aggression. These results were confirmed by the full species-BGLMM (Table 3), although the strength of the relationships changed slightly, particularly for the ones involving mild aggression. Moreover, we obtained very similar results when we analyzed the other four datasets, which used different methods to calculate mild aggression, and one included suspected cased of adulticide (Supplementary Material Table SM2).

**Table 2:** *R* ^2^ values for the five aggression types, calculated from the two null BGLMMs containing the random effect of phylogeny but excluding the five socio-ecological control variables.

Null species-BGLMM: data on the five types of aggression at the species level			
	*R* ^2^ estimate	Estimate error	95% CIs
BG mild aggression	0.22	0.14	0.004–0.518
WG mild aggression	0.09	0.07	0.001–0.250
BG adulticide	0.27	0.11	0.070–0.496
WG adulticide	0.39	0.14	0.114–0.662
Infanticide	0.42	0.11	0.212–0.631
Null sex-BGLMM: data on the three types of lethal aggression divided by sex of the attacker			
	*R* ^2^ estimate	Estimate error	95% CIs
BG female adulticide	0.23	0.15	0.01–0.55
WG female adulticide	0.12	0.15	0.00–0.52
BG male adulticide	0.36	0.13	0.12–0.64
WG male adulticide	0.47	0.15	0.16–0.76
Female infanticide	0.18	0.13	0.01–0.49
Male infanticide	0.59	0.10	0.40–0.78

*Note*. BG = between-group; WG = within-group; and 95% CIs = lower and upper 95% credible intervals.

**Table 3:** Test statistics, of the null and full BGLMMs, for the evolutionary correlation between the five types of aggression at the species level (species-BGLMM) and for the three types of lethal aggression, divided by sex of the attacker (sex-BGLMM).

Species-BGLMM: data on the five types of aggression at the species level								
	Null model				Full model			
	Estimate ± error	95% CIs	%	Tail ESS	Estimate ± error	95% CIs	%	Tail ESS
BG mild aggression—WG mild aggression	−0.14 ± 0.33	−0.72–0.52	66.2	7,526	−0.07 ± 0.32	−0.67–0.56	58.7	7,581
BG mild aggression—BG adulticide	−0.01 ± 0.30	−0.59–0.55	50.7	6,006	0.13 ± 0.29	−0.48–0.65	67.8	2,396
BG mild aggression—WG adulticide	−0.28 ± 0.28	−0.75–0.33	83.5	4,024	−0.16 ± 0.29	−0.68–0.43	71.2	4,258
BG mild aggression—Infanticide	−0.32 ± 0.27	−0.77–0.26	87.9	4,340	−0.07 ± 0.29	−0.61–0.50	60.5	3,680
WG mild aggression—BG adulticide	0.26 ± 0.30	−0.39–0.77	60.8	4,535	0.13 ± 0.30	−0.50–0.67	67.4	3,143
WG mild aggression—WG adulticide	0.09 ± 0.32	−0.54–0.67	61.3	6,212	0.07 ± 0.30	−0.53–0.63	59.6	4,305
WG mild aggression—Infanticide	0.26 ± 0.30	−0.39–0.77	79.8	4,535	0.30 ± 0.30	−0.35–0.79	83.3	3,037
BG adulticide—WG adulticide	0.52 ± 0.22	0.01–0.87	97.7	9,327	0.51 ± 0.21	0.06–0.85	98.6	6,489
BG adulticide—Infanticide	0.44 ± 0.23	−0.05–0.83	96.0	10,225	0.41 ± 0.23	−0.07–0.79	95.5	7,811
WG adulticide—Infanticide	0.48 ± 0.22	−0.02–0.84	97.1	8,425	0.31 ± 0.25	−0.20–0.76	87.9	5,903
Sex-BGLMM: data on the three types of lethal aggression divided by sex of the attacker								
	Null model				Full model			
	Estimate ± error	95% CIs	%	Tail ESS	Estimate ± error	95% CIs	%	Tail ESS
Females								
BG adulticide—WG adulticide	0.24 ± 0.31	−0.43–0.76	78.2	7,350	0.33 ± 0.23	−0.15–0.73	92.0	9,610
BG adulticide—Infanticide	0.11 ± 0.30	−0.50–0.66	64.2	9,689	0.10 ± 0.28	−0.47–0.62	63.9	10,212
WG adulticide—Infanticide	0.10 ± 0.32	−0.53–0.67	62.6	8,747	0.12 ± 0.29	−0.48–0.65	66.8	9,138
Males								
BG adulticide—WG adulticide	0.47 ± 0.21	0.01–0.83	97.6	5,672	0.46 ± 0.18	0.07–0.78	99.0	7,295
BG adulticide—Infanticide	0.49 ± 0.21	0.03–0.83	98.1	9,040	0.43 ± 0.20	0.01–0.77	97.8	7,793
WG adulticide—Infanticide	0.51 ± 0.20	0.08–0.83	98.8	6,168	0.40 ± 0.21	−0.05–0.76	96.0	8,044

*Notes*. Rhat ≤ 1.01 for all the relationships; BG = between-group; WG = within-group; 95% CIs = lower and upper 95% credible intervals; % = percentage of the posterior distribution in the direction of the mean; and Tail ESS = tail effective sample size.

In the two sex-BGLMMs, where we split the data on the three types of lethal aggression by sex of the attacker, the random effect of phylogeny in the null sex-BGLMM explained a greater amount of variance for male than for female killing, and the largest effect was for male infanticide (Table 2). Confirming the results of our species-BGLMMs, we found a strong and positive correlation between the three types of lethal aggression in males in both the null and full sex-BGLMM (Table 3). In females, the relationship between the three types of lethal aggression was weak in the null sex-BGLMM, but was slightly stronger in the full sex-BGLMM; the only strong relationship was for within- and between-group adulticide in the full sex-BGLMM (Table 3). We did not use the four other datasets for the analyses on lethal aggression by sex of the attacker, since these data excluded mild aggression.

## Discussion

We found support for correlated evolution between the three types of lethal aggression, especially in males, but not for mild aggression. We obtained similar results from the five datasets that contained different methods to calculate mild aggression, including one dataset that also considered suspected cases of adulticide. Thus, our results are consistent and not affected by our methodological approach.

Two conclusions can be drawn from our findings. First, there seems to be a clear distinction, among primates, between lethal and mild aggression. Such conclusion may appear intuitive, because these types of aggression bear different payoffs for opponents and have distinct functions (Lukas & Huchard, 2014; Wrangham, 2018). However, animal contest theory predicts that mild aggressive interactions can potentially escalate to lethal aggression, under specific socio-ecological conditions (e.g., high value of the resource at stake or similar fighting abilities for the opponents (Arnott & Elwood, 2009; Hardy & Briffa, 2013). Our results point to a more complex picture: species that display higher frequencies of mild (between- or within-group) aggression do not have a greater potential to kill. Thus, a higher frequency of mild aggression does not necessarily translate into a greater risk of escalation of aggression, because the socio-ecological factors that would trigger such escalation are rarely met in more mildly aggressive species (Aureli et al., 2006; Wrangham, 1999), because killing is too costly or physiologically demanding (Briffa & Sneddon, 2007), or because these species have evolved more effective constraints against the escalation of aggression, such as conflict management strategies (Aureli et al., 2002). We argue that the different occurrence and triggers of non-lethal and lethal aggression should be more formally incorporated into socio-ecological and animal contest models.

Our second conclusion is that extreme caution has to be used when combining together different types of lethal aggression (Dwyer & Micale, 2021; Gómez et al., 2016; Pinker, 2011). Even though we found a positive correlation between the three types of lethal aggression, the strength of the correlation was moderate, and it was weaker for female than for male aggression. Our results conflict with those retrieved by Gómez et al. (2021), who found a positive correlation between adulticide and infanticide in female but not in male mammals. If we apply our findings to analyses on historical rates of aggression (Pinker, 2011), we argue that considering aggression as an undifferentiated trait may hide possible patterns in the occurrence rates of different types of aggression and thus provide inaccurate conclusions on evolutionary or historical trends (Dwyer & Micale, 2021; Ferguson, 2013). Thus, humans and other animal species cannot be described as “indiscriminately” violent (or peaceful); different types of aggression are not all strongly evolutionarily linked, and they can occur under distinct socio-ecological contexts.

Among the taxa represented in our dataset, infanticide was approximately three times more present than adulticide. This difference may be due to the fact that infanticide has been more systematically studied in primates than other types of lethal aggression (Digby, 2000; Lukas & Huchard, 2014, 2019; Opie et al., 2013), or due to the lower risk (for the attackers) of infanticide in comparison to adulticide (Lane & Briffa, 2017; Wrangham, 1999). Moreover, adulticide benefits from cognitively demanding processes: cooperation among attackers, and advance planning about the location of the attack (as in ambushes) and about the number of attackers necessary to outnumber the victim (Wrangham, 1999). Finally, killing conspecific adults is favored by high level of proactive aggression, which is hypothesized to be low in other primates than humans (Wrangham, 2018). Due to these cognitive, neuro-physiological, and behavioral constraints, adulticide in non-human primates (perhaps with the exception of chimpanzees) may mostly occur without planning and thanks to stochastic events, such as the isolation of an individual from another group during an aggressive between-group encounter (Martínez-Íñigo et al., 2021). In our analyses, we could not consider the rates of lethal aggression due to lack of data. Thus, we could not address the question of whether having the potential to kill, but doing so rarely, and killing conspecifics frequently have different adaptive values, evolutionary trajectories, and socio-ecology.

Despite these considerations, around one fifth of the taxa in our dataset displayed either between- or within-group adulticide, which supports the view that lethal aggression is relatively widespread in primates, although not very frequent (Gómez et al., 2016, 2021). The rarity of the occurrence of lethal aggression, and the logistical challenges of observing these events in wild animals, significantly reduce the number of reports of adulticide in the literature. Moreover, the occurrence of adulticide in a species may vary substantially across populations and groups, as observed in chimpanzees (Wilson et al., 2014) and crested macaques (Martínez-Íñigo et al., 2021). Few species have been subject to continuous long-term research on different populations, and only a small portion of species is studied longitudinally (Kappeler & Watts, 2012). Thus, the number of species reported to display adulticide in our data is likely to be a conservative figure.

In summary, our study suggest that primate aggression is not a single trait, and that conspecific killing is weakly related to mild types of aggression. It remains to be determined whether a similar relationship between different types of aggression is also observed in other taxa. Moreover, specific types of aggression, rather than a combination of them all, should be considered when testing hypotheses on social evolution.

## Supplementary Material

qrag002_Supplemental_File

## Data Availability

The data and R code used for this study are available at: https://hdl.handle.net/10779/lincoln.31026160.

## References

[bib1] Arnott G., Elwood R. W. (2009). Assessment of fighting ability in animal contests. Animal Behaviour, 77(5), 991–1004. 10.1016/j.anbehav.2009.02.010

[bib2] Aureli F., Cords M., Van Schaik C. P. (2002). Conflict resolution following aggression in gregarious animals: a predictive framework. Animal Behaviour, 64(3), 325–343. 10.1006/anbe.2002.3071

[bib3] Aureli F., Schaffner C. M., Verpooten J., Slater K., Ramos-Fernandez G. (2006). Raiding parties of male spider monkeys: insights into human warfare?. American Journal of Physical Anthropology, 131(4), 486–497. 10.1002/ajpa.20451 16685723

[bib4] Bowles S. (2012). Warriors, levelers, and the role of conflict in Human social evolution. Science, 336(6083), 876–879. 10.1126/science.1217336 22605768

[bib5] Briffa M., Sneddon L. U. (2007). Physiological constraints on contest behaviour. Functional Ecology, 21(4), 627–637. 10.1111/j.1365-2435.2006.01188.x

[bib6] Bürkner P. (2017a). brms: an R package for bayesian multilevel models using Stan. Journal of Statistical Software, 80, 1–28.

[bib7] Bürkner P. (2017b). Bayesian distributional non-linear multilevel modeling with the R package brms. arXiv, arXiv:1705.11123, preprint. 10.48550/arXiv.1705.11123

[bib8] Cowl V. B., Shultz S. (2017). Large brains and groups associated with high rates of agonism in primates. Behavioral Ecology, 28(3), 803–810. 10.1093/beheco/arx041

[doi65_573_235626] DeCasien A. R., Williams S. A., Higham J. P. (2017). Primate brain size is predicted by diet but not sociality. Nature Ecology & Evolution, 1:(5). 10.1038/s41559-017-0112 28812699

[bib9] Digby L. (2000). Infanticide by female mammals: implications for the evolution of social systems. In Van Schaik C. P., Janson Charlie H. (Eds.), Infanticide by males and its implications(pp. 423–446.). Cambridge University Press. 10.1017/CBO9780511542312

[bib10] Dixson A. F. (1980). Androgens and aggressive behavior in primates: A review. Aggressive Behavior, 6(1), 37–67. 10.1002/1098-2337(1980)6:1<37::AID-AB2480060106>3.0.CO;2-7

[bib11] Dunbar R. I. M., MacCarron P., Robertson C. (2018). Trade-off between fertility and predation risk drives a geometric sequence in the pattern of group sizes in baboons. Biology Letters, 14(3). 10.1098/rsbl.2017.0700 PMC5897608 29514992

[bib12] Dwyer P., Micale M. (2021). The darker angels of our nature: refuting the pinker theory of history & violence. Bloomsbury Publishing. 10.5040/9781350148437

[bib13] Ferguson R. B. (2013). Pinker’s list: exaggerating prehistoric war mortality. In Fry D. P. (Ed.), War, peace, and human nature(pp. 112–131.). Oxford University Press. 10.1093/acprof:oso/9780199858996.001.0001

[bib14] Fry D. P. (2006). The human potential for peace: an anthropological challenge to assumptions about war and violence. Oxford University Press.

[bib15] Gómez J. M., Verdú M., González-Megías A. (2021). Killing conspecific adults in mammals. Proceedings of the Royal Society B, 288(1955), 20211080. 34284635 10.1098/rspb.2021.1080 PMC8292775

[bib16] Gómez J. M., Verdú M., González-Megías A., Méndez M. (2016). The phylogenetic roots of human lethal violence. Nature, 538(7624), 233. 27680701 10.1038/nature19758

[bib17] Grabowski M., Kopperud B. T., Tsuboi M., Hansen T. F. (2023). Both diet and sociality affect primate brain-size evolution. Systematic Biology, 72(2), 404–418. 10.1093/sysbio/syac075 36454664 PMC10275546

[bib18] Hardy I. C., Briffa M. (2013). Animal contests. Cambridge University Press. 10.1017/CBO9781139051248

[bib19] Heldstab S. A., van Schaik C. P., Müller D. W., Rensch E., Lackey L. B., Zerbe P., Hatt J., Clauss M., Matsuda I. (2021). Reproductive seasonality in primates: patterns, concepts and unsolved questions. Biological Reviews, 96(1), 66–88. 10.1111/brv.12646 32964610

[bib20] Hosey G. R. (2005). How does the zoo environment affect the behaviour of captive primates?. Applied Animal Behaviour Science, 90(2), 107–129. 10.1016/j.applanim.2004.08.015

[bib21] Isbell L. A. (1991). Contest and scramble competition: patterns of female aggression and ranging behavior among primates. Behavioral Ecology, 2(2), 143. 10.1093/beheco/2.2.143

[bib22] Kappeler P. M., van Schaik C. P. (2002). Evolution of primate social systems. International Journal of Primatology, 23(4), 707–740. 10.1023/A:1015520830318

[bib23] Kappeler P. M., Watts D. P. (2012). Long-term field studies of primates. Springer Science & Business Media. 10.1007/978-3-642-22514-7

[bib24] Kelly R. L. (2013). From the peaceful to the warlike: ethnographic and archaeological insights into hunter-gatherer warfare and homicide. War, Peace, and Human Nature: The Convergence of Evolutionary and Cultural Views, 151–167. 10.1093/acprof:oso/9780199858996.001.0001

[bib25] Kim N. C., Kissel M. (2018). Emergent warfare in our evolutionary past. Routledge. 10.4324/9781315151021

[bib66_721_230126] Kuhner M. K., Felsenstein J. (1994). A simulation comparison of phylogeny algorithms under equal and unequal evolutionary rates. Mol Biol Evol, 11, 459–68. 8015439 10.1093/oxfordjournals.molbev.a040126

[bib26] Lane S. M., Briffa M. (2017). The price of attack: rethinking damage costs in animal contests. Animal Behaviour, 126, 23–29. 10.1016/j.anbehav.2017.01.015

[bib27] Lemoine S., Boesch C., Preis A., Samuni L., Crockford C., Wittig R. M. (2020). Group dominance increases territory size and reduces neighbour pressure in wild chimpanzees. Royal Society Open Science, 7(5), 200577. 10.1098/rsos.200577 32537232 PMC7277268

[bib28] Lukas D., Huchard E. (2014). The evolution of infanticide by males in mammalian societies. Science, 346(6211), 841–844. 10.1126/science.1257226 25395534

[bib29] Lukas D., Huchard E. (2019). The evolution of infanticide by females in mammals. Philosophical Transactions of the Royal Society B, 374(1780), 20180075. 10.1098/rstb.2018.0075 PMC6664130 31303157

[bib30] Majolo B. (2019). Warfare in an evolutionary perspective. Evolutionary Anthropology, 28(6), 321–331. 10.1002/evan.21806 31691443

[bib31] Majolo B., Ventura R., Koyama N. F. (2005). Sex, rank and age differences in the Japanese Macaque (*Macaca fuscata yakui*) participation in inter-group encounters. Ethology, 111(5), 455–468. 10.1111/j.1439-0310.2005.01087.x

[bib32] Majolo B., Vizioli A. d., Martínez-Íñigo L., Lehmann J. (2020). Effect of group size and individual characteristics on intergroup encounters in primates. International Journal of Primatology, 41(2), 325–341. 10.1007/s10764-019-00119-5

[bib33] Manson J. H., Wrangham R. W., Boone J. L., Chapais B., Dunbar R., Ember C. R., Irons W., Marchant L. F., McGrew W. C., Nishida T. (1991). Intergroup aggression in chimpanzees and humans [and Comments and Replies]. Current Anthropology, 32(4), 369–390. 10.1086/203974

[bib34] Martínez-Íñigo L., Engelhardt A., Agil M., Pilot M., Majolo B. (2021). Intergroup lethal gang attacks in wild crested macaques, Macaca nigra. Animal Behaviour, 180, 81–91.

[bib35] McElreath R. (2020). Statistical rethinking: a bayesian course with examples in r and stan. (2nd ed.). Chapman and Hall/CRC. 10.1201/9780429029608

[bib36] Mitani J. C., Rodman P. S. (1979). Territoriality: the relation of ranging pattern and home range size to defendability, with an analysis of territoriality among primate species. Behavioral Ecology and Sociobiology, 5(3), 241–251. 10.1007/BF00293673

[bib37] Newton-Fisher N. E., Thompson M. E. (2012). Comparative evolutionary perspectives on violence. In Shackelford T. K., Weekes-Shackelford V. A. (Eds.), The oxford handbook of evolutionary perspectives on violence, homicide, and war(pp. 41–60.). Oxford University Press, . 10.1093/oxfordhb/9780199738403.001.0001

[bib38] Opie C., Atkinson Q. D., Dunbar R. I., Shultz S. (2013). Male infanticide leads to social monogamy in primates. Proceedings of the National Academy of Sciences, 110(33), 13328–13332. 10.1073/pnas.1307903110 PMC3746880 23898180

[bib39] Paradis E., Schliep K. (2019). ape 5.0: an environment for modern phylogenetics and evolutionary analyses in R. Bioinformatics, 35(3), 526–528. 10.1093/bioinformatics/bty633 30016406

[bib40] Pinker S. (2011). The better angels of our nature: the decline of violence in history and its causes. Penguin, UK,

[bib41] Plavcan J. M., Van Schaik C. P. (1997). Intrasexual competition and body weight dimorphism in anthropoid primates. American Journal of Physical Anthropology, 103(1), 37–68. 10.1002/(SICI)1096-8644(199705)103:1<37::AID-AJPA4>3.0.CO;2-A 9185951

[bib42] Powell L. E., Isler K., Barton R. A. (2017). Re-evaluating the link between brain size and behavioural ecology in primates. Proceedings of the Royal Society B, 284(1865), 20171765. 10.1098/rspb.2017.1765 29046380 PMC5666103

[bib43] R Core Team . (2020). R: A language and environment for statistical computing [computer software]. R Foundation for Statistical Computing, Vienna.

[bib44] Revell L. J. (2012). phytools: an R package for phylogenetic comparative biology (and other things). Methods in Ecology and Evolution, (2), 217–223. 10.1111/j.2041-210X.2011.00169.x

[bib45] Rowe N., Myers M. (2016). All the world’s primates. Pogonias Press, Charlestown, USA. www.alltheworldsprimates.org

[bib46] Schielzeth H. (2010). Simple means to improve the interpretability of regression coefficients. Methods in Ecology and Evolution, 1(2), 103–113. 10.1111/j.2041-210X.2010.00012.x

[bib47] Schliep K. P. (2011). phangorn: phylogenetic analysis in R. Bioinformatics, 27(4), 592–593. 10.1093/bioinformatics/btq706 21169378 PMC3035803

[bib48] Silk J. B. (2014). The evolutionary roots of lethal conflict. Nature, 513(7518), 321–322. 10.1038/513321a 25230649

[bib49] Smith R. J. (1999). Statistics of sexual size dimorphism. Journal of Human Evolution, 36(4), 423–458. 10.1006/jhev.1998.0281 10208795

[bib50] Stan Development, T . (2024). RStan: the r interface to stan. R package version 2.32.6. Date accessed February 4, 2025. https://mc-stan.org/.

[bib51] Sterck E. H. M., Watts D. P., van Schaik C. P. (1997). The evolution of female social relationships in nonhuman primates. Behavioral Ecology and Sociobiology, 41(5), 291–309. 10.1007/s002650050390

[bib52] Stone A. C., Battistuzzi F. U., Kubatko L. S., Perry G. H. Jr, Trudeau E., Lin H., Kumar S. (2010). More reliable estimates of divergence times in Pan using complete mtDNA sequences and accounting for population structure. Philosophical Transactions of the Royal Society B, 365. 3277–3288. 10.1098/rstb.2010.0096 PMC2981963 20855302

[bib53] Surbeck M., Langergraber K. E., Fruth B., Vigilant L., Hohmann G. (2017). Male reproductive skew is higher in bonobos than chimpanzees. Current Biology, 27(13), R640–R641. 10.1016/j.cub.2017.05.039 28697359 PMC5568700

[bib54] Ting N. (2008). Molecular systematics of red colobus monkeys (Procolobus [Piliocolobus]): understanding the evolution of an endangered primate. City University of New York.

[bib55] Upham N. S., Esselstyn J. A., Jetz W. (2019). Inferring the mammal tree: species-level sets of phylogenies for questions in ecology, evolution, and conservation. PLoS Biology, 17(12), e3000494. 10.1371/journal.pbio.3000494 31800571 PMC6892540

[bib56] Vehtari A., Gelman A., Simpson D., Carpenter B., Bürkner P. (2021). Rank-normalization, folding, and localization: an improved R ^ for assessing convergence of MCMC (with discussion). Bayesian Analysis, 16(2), 667–718. 10.1214/20-BA1221

[bib57] Wheeler B. C., Scarry C. J., Koenig A. (2013). Rates of agonism among female primates: a cross-taxon perspective. Behavioral Ecology, 24(6), 1369–1380. 10.1093/beheco/art076 24137045 PMC3796709

[bib60] Willems E. P., van Schaik C. P. (2015). Collective action and the intensity of between-group competition in nonhuman primates. Behavioral Ecology, 26(2), 625–631. 10.1093/beheco/arv001

[bib58] Willems E. P., Arseneau T. J. M., Schleuning X., van Schaik C. P. (2015). Communal range defence in primates as a public goods dilemma. Philosophical Transactions of the Royal Society B, 370(1683), 20150003. 10.1098/rstb.2015.0003 PMC4633841 26503678

[bib59] Willems E. P., Hellriegel B., van Schaik C. P. (2013). The collective action problem in primate territory economics. Proceedings of the Royal Society B, 280(1759), 20130081. 10.1098/rspb.2013.0081 23516240 PMC3619503

[bib61] Wilson M. L., Boesch C., Fruth B., Furuichi T., Gilby I. C., Hashimoto C., Hobaiter C. L., Hohmann G., Itoh N., Koops K. (2014). Lethal aggression in Pan is better explained by adaptive strategies than human impacts. Nature, 513(7518), 414–417. 10.1038/nature13727 25230664

[bib62] Wrangham R. W. (1999). Evolution of coalitionary killing. American Journal of Physical Anthropology, 110(S29), 1–30. 10.1002/(SICI)1096-8644(1999)110:29+<1::AID-AJPA2>3.0.CO;2-E 10601982

[bib63] Wrangham R. W. (2018). Two types of aggression in human evolution. Proceedings of the National Academy of Sciences of the United States of America, 115(2), 245–253. 10.1073/pnas.1713611115 29279379 PMC5777045

[bib64] Zefferman M. R., Mathew S. (2015). An evolutionary theory of large-scale human warfare: group-structured cultural selection. Evolutionary Anthropology, 24(2), 50–61. 10.1002/evan.21439 25914359

