# Supplementary material for: Origins of violence: evolutionary decoupling between mild and lethal conspecific aggression in primates
Source: Evol Lett. 2026 Mar 3;10(2):207–16. doi: 10.1093/evlett/qrag002 (PMC13043903; doi:10.1093/evlett/qrag002)
Supplement: qrag002_Supplemental_File [file qrag002_supplemental_file.docx]

**Supplementary Material**

**SM1. Data collection**

**SM1.1. Survey data**

We obtained data from the online survey on the following species and sub-species: *Alouatta palliata, Ateles geoffroyi, Brachyteles arachnoides, Callicebus nigrifrons, Cebus capucinus,*

*Cercocebus torquatus, Cercocebus diana, Chlorocebus pygerythrus, Colobus vellerosus, Gorilla gorilla, Macaca assamensis, Macaca fascicularis, Macaca mulatta, Macaca nigra, Macaca sylvanus, Macaca radiata, Pan troglodytes_schweinfurthii Pan troglodytes verus, Papio anubis, Papio cynocephalus, Papio papio, Piliocolobus kirkii, Presbytis thomasi, Propithecus edwardsi, Propithecus diadema, Saguinus labiatus, Theropithecus gelada,*

*Varecia variegata.*

**SM1.2. Survey on between- and within-group aggression and conspecific killing in primates**

Dear researcher,

We are conducting a project on the evolution aggression in non-human primates. This study was approved by the Ethics Committee of the University of Lincoln (reference number: 2020-2393). We are contacting primatologists in order to expand our dataset to include unpublished data, and to increase the chances that we do not miss any relevant published study. For this purpose, we would like to ask you if you could please answer the questions below about your study species. Please note that we are only focusing on non-provisioned populations of primates. If you decide to complete this survey, you will be asked to recall details of aggressive acts that you have witnessed or are aware of. Please do not proceed if you feel this would cause you distress. Moreover, please do not fill in this survey if you feel you don’t have reliable data on aggression in your study species.

Thank you very much for your help!

Q1. We would like to acknowledge all researchers who complete this survey, and therefore ask for you to fill in your name below. If you prefer your responses to remain anonymous then please leave it blank.

________________________________________________________________

Q2. What is the scientific and common name of your study species? Ideally, please use the most recent scientific name. If you have data on multiple populations of the same species, please add the details below. If you have data on more than one species, please fill in the survey separately for each species.

________________________________________________________________

Q3. How long have you studied this species/population(s)? Please either give exact start/end dates or number of years/months.

________________________________________________________________

Q4. What is the location of your study species/population(s)? Please give the name of the country, study site, and/or coordinates.

________________________________________________________________

PART 1 - Lethal Aggression

Q5. Are you aware of lethal within-group and/or between-group lethal aggression in the species you are describing? Based on the definitions given below please select one or more options as appropriate. Please note in this instance lethal aggression does not include infanticide.

Observed = The attack and death of the individual was observed (either by yourself or another researcher).

Inferred = Not directly witnessed but there was clear evidence of lethal aggression; e.g. severe aggression observed but victim escaped and later disappeared or final attack out of sight, dead body was found with clear canine wounds or other skeletal damage consistent with a conspecific attack.

- Observed - Within-group lethal aggression
- Inferred - Within-group lethal aggression
- Observed - Between-group lethal aggression
- Inferred - Between-group lethal aggression
- Not observed

Observed or inferred between-group lethal aggression:

Q6. Please select all combinations of between-group lethal aggression in your study population, in relation to the sex and age of the opponents. Please use your own definition for the different age classes.


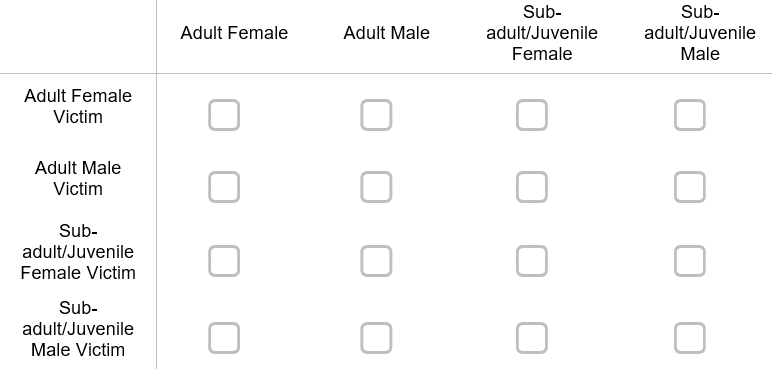


Q7. Please can you provide further contextual information about the inferred lethal aggression (e.g., inferred through bite marks, possible causes, sex of victim and/or aggressor; feel free to write as much as you want)?

________________________________________________________________

Q8. For cases where more than one attacker was observed, were the attackers forming a coalitionary attack against the victim? Coalitionary attack here means that attackers appeared to coordinate their attacks to the victim.

- Yes
- No
- Unsure

Q9. Who was involved in the coalitionary attack? Multiple options can be selected if different lethal events included different coalition demographics.

- Males only
- Females only
- Mixed

Observed or inferred within-group lethal aggression:

Q10. Please select all combinations of within-group lethal aggression in your study population, in relation to the sex and age of the opponents. Please use your own definition for the different age classes.


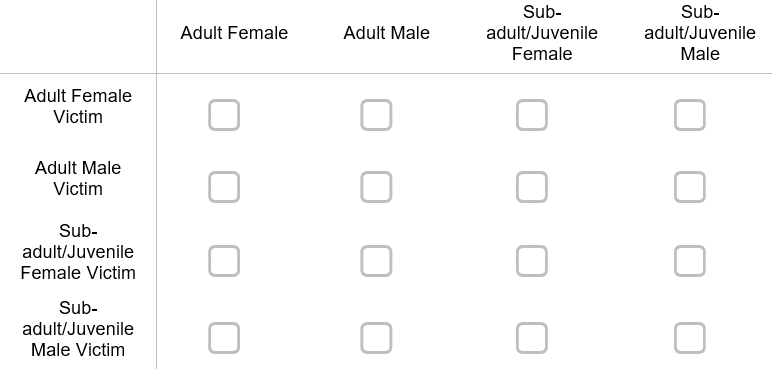


Q11. Please can you provide further contextual information about the inferred lethal aggression (e.g., inferred through bite marks, possible causes, sex of victim and/or aggressor; feel free to write as much as you want)?

________________________________________________________________

Q12. For cases where more than one attacker was observed, were the attackers forming a coalitionary attack against the victim? Coalitionary attack here means that attackers appeared to coordinate their attacks to the victim.

- Yes
- No
- Unsure

Q13. Who was involved in the coalitionary attack? Multiple options can be selected if different lethal events included different coalition demographics.

- Males only
- Females only
- Mixed

PART 2 – Mild aggression

Q14. Have you observed within- or between-group mild aggression in your study species? Mild aggression here means the display of agonistic facial expressions and vocalizations between animals in visual contact (i.e. excluding long-distance aggressive calls advertisement) and/or agonistic physical interactions including chases, pushes, hits, or bites.

- Between-group
- Within-group
- No aggression observed

Between-group mild aggression:

Q15. For the two questions below, please select the relevant sexes in relation to between-group aggression.


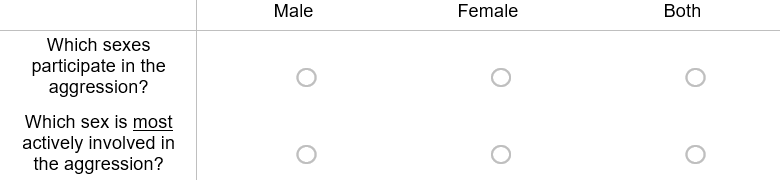


Q16. What is the frequency of this between-group aggression? Please give quantitative data and add details of the unit of measurement (e.g. events/hour/individual); alternatively, please give a qualitative description of the aggression observed for each sex combination (e.g. 'rare' or 'high').

________________________________________________________________

Within-group mild aggression:

Q17. For the two questions below, please select the relevant sexes in relation to within-group aggression.


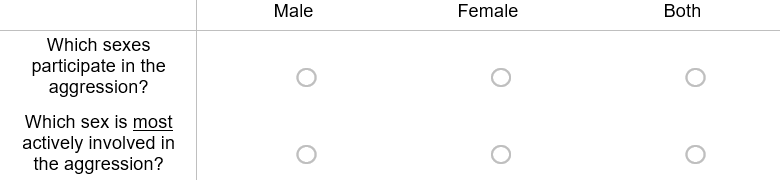


Q18. What is the frequency of this within-group aggression? Please give quantitative data and add details of the unit of measurement (e.g. events/hour/individual); alternatively, please give a qualitative description of the aggression observed for each sex combination (e.g. 'rare' or 'high').

________________________________________________________________

Q19. Please add below the details of any of your publication that presents some of the data on aggression you kindly shared with us.

________________________________________________________________

Thank you very much for completing this survey!

**SM1.3. Conversion of data to the same unit of measurement**

For between- and within-group mild aggression, the units of measurement used in our study were, respectively, the proportion of mildly aggressive intergroup encounters (N of encounters involving mild aggression / total N of encounters recorded during the course of each study) and the frequency of intragroup mild aggression (average N of mild aggression given per individual / hour). When data on between-group mild aggression were provided as percentage of mildly aggressive between-group encounters, we simply converted the percentages to proportions (e.g., 23% became 0.23). For within-group mild aggression, when a study provided data as frequency of aggression per day and per animal, we divided this figure by the total observation time for each focal animal to obtain the frequency of aggression per individual and per hour. When within-group mild aggression was recorded as frequency per group, we divided this figure by the number of adults in the group. We discarded studies that provided data in a format that could not be precisely converted into the units of measurement that we used in our study.

**SM1.4. Conversion of qualitative data to quantitative data**

In the qualitative data included in our study for between-group mild aggression, the proportion of mildly aggressive between-group encounters for a species ranged from being described as ‘none’, ‘never’ or ‘rare’ to a condition where ‘some’, ‘most’ or ‘all’ between-group encounters involved the exchange of mild aggression between members of the opposing groups. The description of within-group mild aggression in the selected qualitative data ranged from ‘none’ or ‘very rare’ to ‘infrequent’ or ‘low’ up to ‘frequent’. Our first step was to identify species for which both qualitative and quantitative data, for between- or within-group mild aggression, were available from the same or from different studies. We used these studies to assign a range of numeric values to each of the qualitative descriptions of mild aggression presented above. So, for example, a ‘rare’ proportion of mildly aggressive between-group encounters was used to describe, in the primary studies, species for which we had quantitative data on between-group mild aggression ranging between 0.18 to 0.43. We applied these numeric ranges to the species for which we only had qualitative data, on between- and/or within-group mild aggression, and we built five different datasets (“Baseline”, “High”, “Low”, “Binary1” and “Binary2”; Table SM1). We used different procedure to integrate qualitative and quantitative data in the five datasets.

In the “High” dataset, we assigned the highest numeric value to the qualitative data, within the range identified from the qualitative and quantitative data. Conversely, in the “Low” dataset, we assigned the lowest numeric value within the range. Thus, for example, in the “High” dataset, we assigned the numeric value of 0.43 to a ‘rare’ proportion of mildly aggressive inter- between-group encounters, whereas in the “Low” dataset we assigned the numeric value of 0.18 to a ‘rare’ proportion of mildly aggressive between-group encounters. In the two binary datasets (“Binary1” and “Binary2”), we calculated the average from the range of numeric values assigned to the qualitative description for that variable and species (e.g., we calculated the average of 0.18 and 0.43 for the species described as having a ‘rare’ proportion of mildly aggressive between-group encounters). We then transformed between- and within-group mild aggression into two binary variables: we assigned the value of zero (i.e., absence) to a species, if their numeric value (from quantitative data or calculated as the average from the range of numeric values assigned to the qualitative data) was < 0.50; we assigned the value of ‘1’ (i.e., presence) to species for which their numeric value was ≥ 0.50. Moreover, in the “Binary#1” dataset we included suspected cases of adulticide (). Finally, in the “Baseline” dataset, we only included quantitative data, when available, for each species and variable; for species for which we had both quantitative and qualitative data, we calculated the average between the quantitative figure and the average of the numeric values (calculated for the two binary datasets).

**Table SM1:** description of the numeric values assigned to the different qualitative data for between- and within-group mild aggression, and criteria used to build the five datasets.

| Dataset | Between-group mild aggression | Within-group mild aggression | Adulticide |
| --- | --- | --- | --- |
| High | ‘None/never’ = 0  ‘Rare’ = 0.43  ‘Some’ = 0.62  ‘Most’ = 0.75  ‘All’ = 1 | ‘None/very rare’ = 0  ‘Rare’ = 0.03  ‘Infrequent/Not frequent’ = 0.21  ‘Low’ = 0.35  ‘Frequent’ = 0.96 | Observed |
| Low | ‘None’ = 0  ‘Rare’ = 0.18  ‘Some’ = 0.28  ‘Most’ = 0.43  ‘All’ = 1 | ‘None/very rare’ = 0  ‘Rare’ = 0.001  ‘Infrequent/Not frequent’ = 0.006  ‘Low’ = 0.034  ‘Frequent’ = 0.96 | Observed |
| Binary1 | ‘Absent’ (< 0.5) = 0  ‘Present’ (≥ 0.5) = 1 | ‘Absent’ (< 0.5) = 0  ‘Present’ (≥ 0.5) = 1 | Observed and suspected |
| Binary2 | ‘Absent’ (< 0.5) = 0  ‘Present’ (≥ 0.5) = 1 | ‘Absent’ (< 0.5) = 0  ‘Present’ (≥ 0.5) = 1 | Observed |
| Baseline | Average | Average | Observed |

**SM2. Data analysis**

In the main text, we present the results of the analyses based on the “Baseline” dataset, because this dataset contains averages for the qualitative data instead of their upper and lower assigned numeric values, and it should thus be the least biased by our data conversion procedures. The assignment of numeric values to qualitative data can be a subjective procedure, as it is based on the researcher’s perception, and it can create biases in comparative analyses (De Block & Vis, 2019). Therefore, we ran additional analyses to assess whether and how our conversion of qualitative data into numeric values affected the results of our study. We ran four Bayesian generalized linear mixed models (BGLMMs) on the four additional datasets (“High”, “Low”, “Binary1” and “Binary2”). We ran these full species-BGLMMs using the same procedure and socio-ecological control variables used for the full species-BGLMM on the “Baseline” dataset presented in the main text. However, for the full species-BGLMMs based on the two binary datasets, we used a Bernoulli distribution for between- and within-group mild aggression, instead of a zero-one inflated beta and a hurdle gamma distribution, because these two response variables were binary-coded in the “Binary1” and “Binary2” datasets.

The results of these BGLMMs (Table SM2) were very similar to what found for the full species-BGLMM based on the “Baseline” dataset (see main text): in all the models, between- and within-group mild aggression were weakly phylogenetically correlated to one another and to the three types of lethal aggression (between- and within-group adulticide, and infanticide). The consistency of the results across the models suggests that our conversion procedure for qualitative data had a negligible effect on our findings.

**Table SM2:** Test statistics for the phylogenetic correlation involving the two types of mild aggression and the three types of lethal aggression, for the three additional datasets (“High”, “Low” and “Binary1” and “Binary2”; see above for details). Rhat = 1 for all the relationships; BG: between-group; WG: within-group; 95%CIs: lower and upper 95% credible intervals; Tail ESS: tail effective sample size.

| “High” dataset | | | |
| --- | --- | --- | --- |
|  | Estimate ± error | 95%CIs | Tail ESS |
| BG mild aggression - WG mild aggression | -0.04 ± 0.34 | -0.67–0.61 | 8,120 |
| BG mild aggression - BG adulticide | 0.08 ± 0.33 | -0.57–0.68 | 3,522 |
| BG mild aggression - WG adulticide | -0.06 ± 0.34 | -0.69–0.61 | 3,727 |
| BG mild aggression - Infanticide | -0.00 ± 0.34 | -0.64–0.65 | 4,176 |
| WG mild aggression - BG adulticide | 0.11 ± 0.31 | -0.51–0.66 | 3,625 |
| WG mild aggression - WG adulticide | 0.07 ± 0.31 | -0.55–0.65 | 5,251 |
| WG mild aggression - Infanticide | 0.17 ± 0.31 | -0.45–0.71 | 4,356 |
| BG adulticide - WG adulticide | 0.51 ± 0.21 | 0.05–0.85 | 7,406 |
| BG adulticide - Infanticide | 0.41 ± 0.23 | -0.07–0.79 | 7,698 |
| WG adulticide - Infanticide | 0.32 ± 0.25 | -0.22–0.77 | 6,810 |
| “Low” dataset | | | |
|  | Estimate ± error | 95%CIs | Tail ESS |
| BG mild aggression - WG mild aggression | -0.07 ± 0.33 | -0.68–0.57 | 8,928 |
| BG mild aggression - BG adulticide | 0.08 ± 0.32 | -0.55–0.66 | 3,968 |
| BG mild aggression - WG adulticide | -0.12 ± 0.34 | -0.72–0.57 | 7,713 |
| BG mild aggression - Infanticide | -0.06 ± 0.31 | -0.65–0.56 | 3,571 |
| WG mild aggression - BG adulticide | 0.10 ± 0.31 | -0.54–0.66 | 3,583 |
| WG mild aggression - WG adulticide | 0.02 ± 0.34 | -0.63–0.65 | 7,893 |
| WG mild aggression - Infanticide | 0.25 ± 0.31 | -0.42–0.77 | 3,328 |
| BG adulticide - WG adulticide | 0.34 ± 0.31 | -0.38–0.82 | 2,353 |
| BG adulticide - Infanticide | 0.44 ± 0.22 | -0.05–0.81 | 8,079 |
| WG adulticide - Infanticide | 0.18 ± 0.22 | -0.50–0.73 | 3,253 |
| “Binary1” dataset | | | |
|  | Estimate ± error | 95%CIs | Tail ESS |
| BG mild aggression - WG mild aggression | 0.10 ± 0.30 | -0.53–0.64 | 4,830 |
| BG mild aggression - BG adulticide | 0.12 ± 0.29 | -0.45–0.67 | 5,034 |
| BG mild aggression - WG adulticide | 0.03 ± 0.31 | -0.55–0.62 | 4,964 |
| BG mild aggression - Infanticide | 0.19 ± 0.28 | -0.38–0.70 | 5,254 |
| WG mild aggression - BG adulticide | 0.00 ± 0.27 | -0.53–0.52 | 7,337 |
| WG mild aggression - WG adulticide | 0.10 ± 0.29 | -0.48–0.64 | 3,240 |
| WG mild aggression - Infanticide | 0.19 ± 0.27 | -0.38–0.67 | 5,333 |
| BG adulticide - WG adulticide | 0.44 ± 0.24 | -0.07–0.83 | 7,723 |
| BG adulticide - Infanticide | 0.40 ± 0.25 | -0.14–0.83 | 7,771 |
| WG adulticide - Infanticide | 0.32 ± 0,26 | -0.23–0.78 | 5,882 |
| “Binary2” dataset |  |  |  |
|  | Estimate ± error | 95%CIs | Tail ESS |
| BG mild aggression - WG mild aggression | 0.22 ± 0.27 | -0.36–0.70 | 3,304 |
| BG mild aggression - BG adulticide | 0.37 ± 0.25 | -0.20–0.77 | 3,389 |
| BG mild aggression - WG adulticide | -0.02 ± 0.32 | -0.62–0.61 | 8,621 |
| BG mild aggression - Infanticide | 0.10 ± 0.28 | -0.46–0.61 | 4,109 |
| WG mild aggression - BG adulticide | 0.28 ± 0.22 | -0.18–0.66 | 6,902 |
| WG mild aggression - WG adulticide | 0.00 ± 0.31 | -0.60–0.61 | 7,925 |
| WG mild aggression - Infanticide | 0.36 ± 0.21 | -0.11–0.73 | 5,355 |
| BG adulticide - WG adulticide | 0.33 ± 0.30 | -0.36–0.80 | 3,884 |
| BG adulticide - Infanticide | 0.47 ± 0.21 | 0.01–0.82 | 8,775 |
| WG adulticide - Infanticide | 0.16 ± 0.32 | -0.49–0.72 | 3,685 |

**References**

De Block, D., & Vis, B. (2019). Addressing the challenges related to transforming qualitative into quantitative data in qualitative comparative analysis. *Journal of Mixed Methods Research*, 13(4), 503-535.

**Results of the models presented in the manuscript**

**1. Results of the null species-BGLMM on the five types of aggression**

Family: MV(gaussian, gaussian, bernoulli, bernoulli, bernoulli)

Links: mu = identity; sigma = identity

mu = identity; sigma = identity

mu = logit

mu = logit

mu = logit

Formula: ba.OG_Agg ~ (1 | p | gr(SpeciesTree, cov = A))

ba.IG_Agg ~ (1 | p | gr(SpeciesTree, cov = A))

ba.IG_Kill ~ (1 | p | gr(SpeciesTree, cov = A))

ba.OG_Kill ~ (1 | p | gr(SpeciesTree, cov = A))

ba.Inf_Kill ~ (1 | p | gr(SpeciesTree, cov = A))

Data: data1 (Number of observations: 100)

Draws: 4 chains, each with iter = 6000; warmup = 3000; thin = 1;

total post-warmup draws = 12000

Multilevel Hyperparameters:

~SpeciesTree (Number of levels: 100)

Estimate Est.Error l-95% CI u-95% CI Rhat Bulk_ESS Tail_ESS

sd(baOGAgg_Intercept) 0.04 0.02 0.00 0.09 1.00 1338 1968

sd(baIGAgg_Intercept) 0.02 0.01 0.00 0.05 1.00 4429 4117

sd(baIGKill_Intercept) 0.67 0.36 0.22 1.60 1.00 3827 5375

sd(baOGKill_Intercept) 0.40 0.20 0.13 0.88 1.00 4646 6235

sd(baInfKill_Intercept) 0.49 0.24 0.20 1.10 1.00 4305 5287

cor(baOGAgg_Intercept,baIGAgg_Intercept) -0.14 0.33 -0.72 0.52 1.00 9441 7526

cor(baOGAgg_Intercept,baIGKill_Intercept) -0.28 0.28 -0.75 0.33 1.00 3298 4024

cor(baIGAgg_Intercept,baIGKill_Intercept) 0.09 0.32 -0.54 0.67 1.00 3091 6212

cor(baOGAgg_Intercept,baOGKill_Intercept) -0.01 0.30 -0.59 0.55 1.00 3906 6006

cor(baIGAgg_Intercept,baOGKill_Intercept) 0.08 0.31 -0.53 0.66 1.00 3508 6117

cor(baIGKill_Intercept,baOGKill_Intercept) 0.52 0.22 0.01 0.87 1.00 7830 9327

cor(baOGAgg_Intercept,baInfKill_Intercept) -0.32 0.27 -0.77 0.26 1.00 3676 4340

cor(baIGAgg_Intercept,baInfKill_Intercept) 0.26 0.30 -0.39 0.77 1.00 3245 4535

cor(baIGKill_Intercept,baInfKill_Intercept) 0.48 0.22 -0.02 0.84 1.00 6594 8425

cor(baOGKill_Intercept,baInfKill_Intercept) 0.44 0.23 -0.05 0.83 1.00 7216 10225

Regression Coefficients:

Estimate Est.Error l-95% CI u-95% CI Rhat Bulk_ESS Tail_ESS

baOGAgg_Intercept 0.49 0.23 0.03 0.96 1.00 4991 4812

baIGAgg_Intercept 0.19 0.14 -0.10 0.49 1.00 6224 6774

baIGKill_Intercept -1.60 2.17 -6.23 2.47 1.00 7815 6659

baOGKill_Intercept -1.43 1.53 -4.64 1.56 1.00 8043 6965

baInfKill_Intercept 0.91 1.72 -2.47 4.46 1.00 8570 8037

Further Distributional Parameters:

Estimate Est.Error l-95% CI u-95% CI Rhat Bulk_ESS Tail_ESS

sigma_baOGAgg 0.32 0.04 0.25 0.39 1.00 2026 3462

sigma_baIGAgg 0.40 0.03 0.35 0.47 1.00 12359 8835

Draws were sampled using sampling(NUTS). For each parameter, Bulk_ESS

and Tail_ESS are effective sample size measures, and Rhat is the potential

scale reduction factor on split chains (at convergence, Rhat = 1).

Estimate Est.Error Q2.5 Q97.5

R2baOGAgg 0.2202088 0.13980393 0.004756519 0.5180978

R2baIGAgg 0.0936214 0.06857504 0.001218608 0.2509668

R2baIGKill 0.3876394 0.13935342 0.114404735 0.6621773

R2baOGKill 0.2761501 0.10948172 0.069623188 0.4954871

R2baInfKill 0.4186307 0.10484765 0.212842779 0.6316331

**2. Results of the full species-BGLMM on the five types of aggression**

Family: MV(gaussian, gaussian, bernoulli, bernoulli, bernoulli)

Links: mu = identity; sigma = identity

mu = identity; sigma = identity

mu = logit

mu = logit

mu = logit

Formula: ba.OG_Agg ~ z.D_Index + z.Group_Size + z.Ldimorphism + z.MF_Ratio + DietCat + (1 | p | gr(SpeciesTree, cov = A))

ba.IG_Agg ~ z.D_Index + z.Group_Size + z.Ldimorphism + z.MF_Ratio + DietCat + (1 | p | gr(SpeciesTree, cov = A))

ba.IG_Kill ~ z.D_Index + z.Group_Size + z.Ldimorphism + z.MF_Ratio + DietCat + (1 | p | gr(SpeciesTree, cov = A))

ba.OG_Kill ~ z.D_Index + z.Group_Size + z.Ldimorphism + z.MF_Ratio + DietCat + (1 | p | gr(SpeciesTree, cov = A))

ba.Inf_Kill ~ z.D_Index + z.Group_Size + z.Ldimorphism + z.MF_Ratio + DietCat + (1 | p | gr(SpeciesTree, cov = A))

Data: data1 (Number of observations: 100)

Draws: 4 chains, each with iter = 6000; warmup = 3000; thin = 1;

total post-warmup draws = 12000

Multilevel Hyperparameters:

~SpeciesTree (Number of levels: 100)

Estimate Est.Error l-95% CI u-95% CI Rhat Bulk_ESS Tail_ESS

sd(baOGAgg_Intercept) 0.04 0.02 0.01 0.08 1.00 1550 2411

sd(baIGAgg_Intercept) 0.03 0.02 0.00 0.07 1.00 1993 2569

sd(baIGKill_Intercept) 1.19 0.92 0.28 3.75 1.00 1302 1602

sd(baOGKill_Intercept) 1.70 1.16 0.35 4.59 1.01 1218 2778

sd(baInfKill_Intercept) 1.65 1.23 0.23 4.64 1.00 906 2469

cor(baOGAgg_Intercept,baIGAgg_Intercept) -0.07 0.32 -0.67 0.56 1.00 6176 7581

cor(baOGAgg_Intercept,baIGKill_Intercept) -0.16 0.29 -0.68 0.43 1.00 2510 4258

cor(baIGAgg_Intercept,baIGKill_Intercept) 0.07 0.30 -0.53 0.63 1.00 2098 4305

cor(baOGAgg_Intercept,baOGKill_Intercept) 0.13 0.29 -0.48 0.65 1.00 1457 2396

cor(baIGAgg_Intercept,baOGKill_Intercept) 0.13 0.30 -0.50 0.67 1.01 1651 3143

cor(baIGKill_Intercept,baOGKill_Intercept) 0.51 0.21 0.06 0.85 1.00 4053 6489

cor(baOGAgg_Intercept,baInfKill_Intercept) -0.07 0.29 -0.61 0.50 1.00 1723 3680

cor(baIGAgg_Intercept,baInfKill_Intercept) 0.30 0.30 -0.35 0.79 1.00 1610 3037

cor(baIGKill_Intercept,baInfKill_Intercept) 0.31 0.25 -0.20 0.76 1.00 2671 5903

cor(baOGKill_Intercept,baInfKill_Intercept) 0.41 0.23 -0.07 0.79 1.00 4560 7811

Regression Coefficients:

Estimate Est.Error l-95% CI u-95% CI Rhat Bulk_ESS Tail_ESS

baOGAgg_Intercept 0.46 0.21 0.02 0.89 1.00 3205 4038

baIGAgg_Intercept 0.20 0.18 -0.19 0.56 1.00 4037 5394

baIGKill_Intercept -1.25 2.74 -7.05 3.89 1.00 5240 4049

baOGKill_Intercept -2.88 3.22 -10.39 2.50 1.00 6112 4598

baInfKill_Intercept -0.70 3.35 -7.96 5.69 1.00 3365 5082

baOGAgg_z.D_Index 0.06 0.04 -0.02 0.14 1.00 10036 8652

baOGAgg_z.Group_Size -0.02 0.04 -0.11 0.07 1.00 8287 8915

baOGAgg_z.Ldimorphism -0.07 0.04 -0.16 0.01 1.00 11539 9243

baOGAgg_z.MF_Ratio 0.02 0.05 -0.08 0.12 1.00 8308 8400

baOGAgg_DietCat -0.05 0.10 -0.26 0.15 1.00 8124 7698

baIGAgg_z.D_Index 0.03 0.05 -0.06 0.13 1.00 9454 7938

baIGAgg_z.Group_Size 0.00 0.05 -0.10 0.10 1.00 10264 9053

baIGAgg_z.Ldimorphism -0.02 0.05 -0.12 0.08 1.00 12094 8956

baIGAgg_z.MF_Ratio -0.01 0.06 -0.13 0.10 1.00 9658 9037

baIGAgg_DietCat -0.08 0.12 -0.30 0.15 1.00 9374 9497

baIGKill_z.D_Index -1.51 1.36 -4.78 0.56 1.00 4420 2935

baIGKill_z.Group_Size 1.62 1.13 0.12 4.55 1.00 3323 2047

baIGKill_z.Ldimorphism -1.05 1.30 -4.11 0.90 1.00 5038 3223

baIGKill_z.MF_Ratio -1.85 1.66 -6.07 0.34 1.00 2993 2419

baIGKill_DietCat 0.62 2.79 -3.73 7.34 1.00 3275 2085

baOGKill_z.D_Index -2.04 2.19 -7.90 0.65 1.00 2912 3099

baOGKill_z.Group_Size 0.66 1.19 -1.38 3.45 1.00 5754 3820

baOGKill_z.Ldimorphism -1.63 1.97 -6.59 1.00 1.00 3382 2463

baOGKill_z.MF_Ratio -1.52 1.81 -6.06 1.12 1.00 3334 2825

baOGKill_DietCat 5.93 5.53 -0.24 20.30 1.00 1890 2467

baInfKill_z.D_Index -1.25 1.30 -4.67 0.37 1.00 2011 2854

baInfKill_z.Group_Size 3.35 2.81 0.19 10.51 1.00 1414 3211

baInfKill_z.Ldimorphism -0.32 1.23 -3.40 1.84 1.00 4736 2667

baInfKill_z.MF_Ratio -0.96 1.43 -4.48 1.29 1.00 4173 3343

baInfKill_DietCat 6.93 5.57 0.78 21.83 1.00 1534 3043

Further Distributional Parameters:

Estimate Est.Error l-95% CI u-95% CI Rhat Bulk_ESS Tail_ESS

sigma_baOGAgg 0.33 0.03 0.26 0.39 1.00 2429 2892

sigma_baIGAgg 0.40 0.03 0.34 0.48 1.00 6677 7979

Draws were sampled using sampling(NUTS). For each parameter, Bulk_ESS

and Tail_ESS are effective sample size measures, and Rhat is the potential

scale reduction factor on split chains (at convergence, Rhat = 1).

Estimate Est.Error Q2.5 Q97.5

R2baOGAgg 0.2665278 0.1068562 0.08934165 0.4994111

R2baIGAgg 0.1655580 0.0782680 0.03833914 0.3333217

R2baIGKill 0.5508720 0.1430599 0.29492954 0.8504403

R2baOGKill 0.6069434 0.1594207 0.29946360 0.8859041

R2baInfKill 0.6823530 0.1510913 0.37980796 0.9238120

**3. Results of the null sex-BGLMM on the three types of lethal aggression, divided by sex of the attacker**

Family: MV(bernoulli, bernoulli, bernoulli, bernoulli, bernoulli, bernoulli)

Links: mu = logit

mu = logit

mu = logit

mu = logit

mu = logit

mu = logit

Formula: IG_Kill_M ~ (1 | p | gr(SpeciesTree, cov = A))

OG_Kill_M ~ (1 | p | gr(SpeciesTree, cov = A))

IG_Kill_F ~ (1 | p | gr(SpeciesTree, cov = A))

OG_Kill_F ~ (1 | p | gr(SpeciesTree, cov = A))

Inf_Kill_M ~ (1 | p | gr(SpeciesTree, cov = A))

Inf_Kill_F ~ (1 | p | gr(SpeciesTree, cov = A))

Data: data2 (Number of observations: 100)

Draws: 4 chains, each with iter = 6000; warmup = 3000; thin = 1;

total post-warmup draws = 12000

Multilevel Hyperparameters:

~SpeciesTree (Number of levels: 100)

Estimate Est.Error l-95% CI u-95% CI Rhat Bulk_ESS

sd(IGKillM_Intercept) 0.89 0.48 0.28 2.14 1.00 2844

sd(OGKillM_Intercept) 0.57 0.34 0.18 1.48 1.00 3063

sd(IGKillF_Intercept) 0.37 0.38 0.01 1.44 1.00 2669

sd(OGKillF_Intercept) 0.51 0.35 0.07 1.41 1.00 2792

sd(InfKillM_Intercept) 0.80 0.38 0.33 1.81 1.00 4462

sd(InfKillF_Intercept) 0.31 0.24 0.03 0.95 1.00 2539

cor(IGKillM_Intercept,OGKillM_Intercept) 0.47 0.21 0.01 0.83 1.00 4707

cor(IGKillM_Intercept,IGKillF_Intercept) 0.31 0.31 -0.37 0.80 1.00 8031

cor(OGKillM_Intercept,IGKillF_Intercept) 0.21 0.30 -0.43 0.74 1.00 10116

cor(IGKillM_Intercept,OGKillF_Intercept) 0.37 0.25 -0.18 0.80 1.00 7065

cor(OGKillM_Intercept,OGKillF_Intercept) 0.56 0.24 -0.03 0.90 1.00 5489

cor(IGKillF_Intercept,OGKillF_Intercept) 0.24 0.31 -0.43 0.76 1.00 6329

cor(IGKillM_Intercept,InfKillM_Intercept) 0.51 0.20 0.08 0.83 1.00 4105

cor(OGKillM_Intercept,InfKillM_Intercept) 0.49 0.21 0.03 0.83 1.00 5618

cor(IGKillF_Intercept,InfKillM_Intercept) 0.11 0.30 -0.48 0.68 1.00 4576

cor(OGKillF_Intercept,InfKillM_Intercept) 0.30 0.26 -0.25 0.75 1.00 4925

cor(IGKillM_Intercept,InfKillF_Intercept) 0.23 0.29 -0.38 0.72 1.00 7408

cor(OGKillM_Intercept,InfKillF_Intercept) 0.09 0.29 -0.48 0.63 1.00 9559

cor(IGKillF_Intercept,InfKillF_Intercept) 0.10 0.32 -0.53 0.67 1.00 6870

cor(OGKillF_Intercept,InfKillF_Intercept) 0.11 0.30 -0.50 0.66 1.00 8541

cor(InfKillM_Intercept,InfKillF_Intercept) 0.10 0.28 -0.44 0.61 1.00 11716

Tail_ESS

sd(IGKillM_Intercept) 3432

sd(OGKillM_Intercept) 4637

sd(IGKillF_Intercept) 4702

sd(OGKillF_Intercept) 3237

sd(InfKillM_Intercept) 7141

sd(InfKillF_Intercept) 4170

cor(IGKillM_Intercept,OGKillM_Intercept) 5762

cor(IGKillM_Intercept,IGKillF_Intercept) 8635

cor(OGKillM_Intercept,IGKillF_Intercept) 8990

cor(IGKillM_Intercept,OGKillF_Intercept) 5499

cor(OGKillM_Intercept,OGKillF_Intercept) 5614

cor(IGKillF_Intercept,OGKillF_Intercept) 7350

cor(IGKillM_Intercept,InfKillM_Intercept) 6168

cor(OGKillM_Intercept,InfKillM_Intercept) 9040

cor(IGKillF_Intercept,InfKillM_Intercept) 6360

cor(OGKillF_Intercept,InfKillM_Intercept) 6796

cor(IGKillM_Intercept,InfKillF_Intercept) 7774

cor(OGKillM_Intercept,InfKillF_Intercept) 8922

cor(IGKillF_Intercept,InfKillF_Intercept) 8747

cor(OGKillF_Intercept,InfKillF_Intercept) 9689

cor(InfKillM_Intercept,InfKillF_Intercept) 10022

Regression Coefficients:

Estimate Est.Error l-95% CI u-95% CI Rhat Bulk_ESS Tail_ESS

IGKillM_Intercept -1.10 2.31 -5.85 3.39 1.00 5929 6945

OGKillM_Intercept -0.87 1.79 -4.45 2.73 1.00 6377 7202

IGKillF_Intercept -2.78 1.74 -6.33 0.85 1.00 5254 5121

OGKillF_Intercept -2.20 1.92 -6.21 1.53 1.00 6856 7325

InfKillM_Intercept 1.07 2.22 -3.21 5.80 1.00 8990 6910

InfKillF_Intercept -1.10 1.37 -3.70 1.93 1.00 6226 5711

Draws were sampled using sampling(NUTS). For each parameter, Bulk_ESS

and Tail_ESS are effective sample size measures, and Rhat is the potential

scale reduction factor on split chains (at convergence, Rhat = 1).

Estimate Est.Error Q2.5 Q97.5

R2IGKillM 0.4654202 0.15353440 0.1613221685 0.7585248

R2OGKillM 0.3552070 0.13169072 0.1153556267 0.6431443

R2IGKillF 0.1150971 0.14461680 0.0001192062 0.5222533

R2OGKillF 0.2302520 0.14924443 0.0079048867 0.5555038

R2InfKillM 0.5888160 0.09877534 0.3950229185 0.7836706

R2InfKillF 0.1815771 0.13174612 0.0028781424 0.4856320

**4. Results of the full sex-BGLMM on the three types of lethal aggression, divided by sex of the attacker**

Family: MV(bernoulli, bernoulli, bernoulli, bernoulli, bernoulli, bernoulli)

Links: mu = logit

mu = logit

mu = logit

mu = logit

mu = logit

mu = logit

Formula: IG_Kill_M ~ z.D_Index + z.Group_Size + z.Ldimorphism + z.MF_Ratio + DietCat + (1 | p | gr(SpeciesTree, cov = A))

OG_Kill_M ~ z.D_Index + z.Group_Size + z.Ldimorphism + z.MF_Ratio + DietCat + (1 | p | gr(SpeciesTree, cov = A))

IG_Kill_F ~ z.D_Index + z.Group_Size + z.Ldimorphism + z.MF_Ratio + DietCat + (1 | p | gr(SpeciesTree, cov = A))

OG_Kill_F ~ z.D_Index + z.Group_Size + z.Ldimorphism + z.MF_Ratio + DietCat + (1 | p | gr(SpeciesTree, cov = A))

Inf_Kill_M ~ z.D_Index + z.Group_Size + z.Ldimorphism + z.MF_Ratio + DietCat + (1 | p | gr(SpeciesTree, cov = A))

Inf_Kill_F ~ z.D_Index + z.Group_Size + z.Ldimorphism + z.MF_Ratio + DietCat + (1 | p | gr(SpeciesTree, cov = A))

Data: data2 (Number of observations: 100)

Draws: 4 chains, each with iter = 6000; warmup = 3000; thin = 1;

total post-warmup draws = 12000

Multilevel Hyperparameters:

~SpeciesTree (Number of levels: 100)

Estimate Est.Error l-95% CI u-95% CI Rhat Bulk_ESS Tail_ESS

sd(IGKillM_Intercept) 2.02 1.20 0.47 4.93 1.00 3037 5757

sd(OGKillM_Intercept) 2.52 1.23 0.70 5.40 1.00 3967 4501

sd(IGKillF_Intercept) 2.59 1.31 0.43 5.53 1.00 3930 3488

sd(OGKillF_Intercept) 1.64 1.06 0.35 4.36 1.00 3574 5779

sd(InfKillM_Intercept) 1.99 1.16 0.43 4.82 1.00 2792 5232

sd(InfKillF_Intercept) 0.73 0.92 0.06 3.58 1.00 1152 1527

cor(IGKillM_Intercept,OGKillM_Intercept) 0.46 0.18 0.07 0.78 1.00 4519 7295

cor(IGKillM_Intercept,IGKillF_Intercept) 0.46 0.21 -0.00 0.80 1.00 7757 8540

cor(OGKillM_Intercept,IGKillF_Intercept) 0.27 0.22 -0.19 0.67 1.00 9283 9883

cor(IGKillM_Intercept,OGKillF_Intercept) 0.37 0.22 -0.08 0.77 1.00 6402 7301

cor(OGKillM_Intercept,OGKillF_Intercept) 0.70 0.16 0.33 0.93 1.00 9340 8613

cor(IGKillF_Intercept,OGKillF_Intercept) 0.33 0.23 -0.15 0.73 1.00 10318 9610

cor(IGKillM_Intercept,InfKillM_Intercept) 0.40 0.21 -0.05 0.76 1.00 4440 8044

cor(OGKillM_Intercept,InfKillM_Intercept) 0.43 0.20 0.01 0.77 1.00 5324 7793

cor(IGKillF_Intercept,InfKillM_Intercept) -0.03 0.24 -0.50 0.45 1.00 6118 7667

cor(OGKillF_Intercept,InfKillM_Intercept) 0.22 0.24 -0.28 0.68 1.00 5000 7848

cor(IGKillM_Intercept,InfKillF_Intercept) 0.22 0.28 -0.37 0.70 1.00 8939 8865

cor(OGKillM_Intercept,InfKillF_Intercept) 0.13 0.26 -0.41 0.62 1.00 11412 9200

cor(IGKillF_Intercept,InfKillF_Intercept) 0.12 0.29 -0.48 0.65 1.00 6782 9138

cor(OGKillF_Intercept,InfKillF_Intercept) 0.10 0.28 -0.47 0.62 1.00 9179 10212

cor(InfKillM_Intercept,InfKillF_Intercept) 0.12 0.28 -0.43 0.65 1.00 10080 10747

Regression Coefficients:

Estimate Est.Error l-95% CI u-95% CI Rhat Bulk_ESS Tail_ESS

IGKillM_Intercept -0.63 3.11 -7.01 5.67 1.00 11411 7484

OGKillM_Intercept -2.99 3.53 -10.55 3.41 1.00 11825 8114

IGKillF_Intercept -0.75 5.23 -12.84 8.01 1.00 6080 4310

OGKillF_Intercept -2.28 3.09 -9.14 3.18 1.00 13635 6926

InfKillM_Intercept -1.54 3.58 -8.82 5.12 1.00 7136 7096

InfKillF_Intercept -0.99 2.08 -5.01 3.41 1.00 9864 5036

IGKillM_z.D_Index -1.73 2.01 -6.70 1.49 1.00 8291 6734

IGKillM_z.Group_Size 2.95 1.89 0.50 7.80 1.00 5551 5343

IGKillM_z.Ldimorphism 0.01 1.83 -3.65 4.07 1.00 8760 6588

IGKillM_z.MF_Ratio -0.84 2.00 -5.62 2.74 1.00 7484 6097

IGKillM_DietCat 2.20 4.19 -4.56 12.53 1.00 7137 5153

OGKillM_z.D_Index -2.20 2.48 -8.21 1.48 1.00 7736 7207

OGKillM_z.Group_Size 0.46 1.49 -2.50 3.79 1.00 9286 7889

OGKillM_z.Ldimorphism -0.74 2.08 -5.47 3.16 1.00 8486 7047

OGKillM_z.MF_Ratio -1.88 2.24 -7.20 1.77 1.00 8353 7301

OGKillM_DietCat 10.84 7.06 1.41 28.74 1.00 5743 6692

IGKillF_z.D_Index -14.36 9.70 -38.48 -2.06 1.00 4666 4940

IGKillF_z.Group_Size -2.89 3.29 -11.26 1.62 1.00 6909 6360

IGKillF_z.Ldimorphism -2.83 3.13 -10.62 1.61 1.00 7254 6613

IGKillF_z.MF_Ratio -4.75 4.18 -14.74 1.33 1.00 5193 5420

IGKillF_DietCat -6.62 7.37 -24.38 5.51 1.00 6072 4836

OGKillF_z.D_Index -1.49 1.88 -5.82 1.77 1.00 9563 6387

OGKillF_z.Group_Size 1.03 1.35 -1.00 4.45 1.00 7247 5692

OGKillF_z.Ldimorphism -2.22 1.99 -7.12 0.75 1.00 9172 6393

OGKillF_z.MF_Ratio -1.56 1.69 -5.21 1.60 1.00 10034 7119

OGKillF_DietCat 2.44 3.78 -3.03 12.26 1.00 7298 5798

InfKillM_z.D_Index -1.93 1.50 -5.74 0.06 1.00 5858 6180

InfKillM_z.Group_Size 4.80 3.36 0.68 13.51 1.00 4102 5628

InfKillM_z.Ldimorphism 0.35 1.25 -2.11 3.10 1.00 9608 6292

InfKillM_z.MF_Ratio -1.45 1.67 -5.31 1.55 1.00 10072 6593

InfKillM_DietCat 8.69 6.05 1.21 24.20 1.00 4238 6090

InfKillF_z.D_Index -0.17 0.79 -1.65 1.54 1.00 7144 3103

InfKillF_z.Group_Size 1.03 0.85 -0.14 3.20 1.00 3882 1851

InfKillF_z.Ldimorphism -1.03 1.34 -4.72 0.54 1.00 4294 2111

InfKillF_z.MF_Ratio 0.36 0.94 -1.50 2.21 1.00 7750 2616

InfKillF_DietCat -0.54 1.93 -3.74 4.06 1.00 5462 2598

Draws were sampled using sampling(NUTS). For each parameter, Bulk_ESS

and Tail_ESS are effective sample size measures, and Rhat is the potential

scale reduction factor on split chains (at convergence, Rhat = 1).

Estimate Est.Error Q2.5 Q97.5

R2IGKillM 0.6784631 0.1376522 0.39896886 0.9157439

R2OGKillM 0.7361336 0.1213207 0.45887144 0.9246810

R2IGKillF 0.6788209 0.1969793 0.20385657 0.9786606

R2OGKillF 0.5357168 0.1615602 0.21059741 0.8425574

R2InfKillM 0.7829653 0.1003238 0.56794921 0.9427079

R2InfKillF 0.3454992 0.1916623 0.08625099 0.8127354
